# News and Notes

**Published:** 1904-12

**Authors:** 


### NEWS AND NOTES.

A SOCIETY for the suppression of dust on streets has been founded in Munich.

A LARGE hospital is to be erected in Philadelphia for the study of cancer and allied diseases.

THE new surgical building at Johns Hopkins Hospital was dedicated with elaborate ceremonies October 5.

NEW ORLEANS is to be the site of a hospital built by the United States government for Panama canal patients.

THE State Board of Health has adopted stricter rules, requiring examination of physicians of other States desiring to practice in Kentucky.

PROFESSOR ORTH, the Kaiser's surgeon, is making a tour of the United States. He is considered one of the most eminent pathologists in Europe.

The Medical Directory of the city of New York, in the edition of 1904 just issued, contains the names of 5,909 practitioners in Greater New York.

DR. WILLIAM LORD SMITH, of Worcester, Mass., according to a report from that city, has received the honorary appointment of physician in ordinary to the Shah of Persia.

SPITTING on pavements will in future be a costly luxury in Seneca, Ill. An ordinance, recently passed by the city council, makes the offense punishable by a fine of $3 to $25.

---

DR. ERICH BENNECKE, extraordinary professor of surgery in the University of Berlin, died recently in the fortieth year of his age of septicemia contracted in the course of an operation.

---

IT is stated that the government of the Mikado intends to introduce on a large scale into Japan the system of protection against malaria which has been so successful in Italy and elsewhere.

---

HEALTH OFFICER CRUM, of Ithaca, N. Y., has asked help from the State Health Department to control a threatened epidemic of rabies which has been developing there for the last two weeks.

---

THE New York Post-Graduate School and Hospital has been given an adjoining house, which is to be converted into a hospital for the treatment of consumptives, and has been promised $6,000 yearly for its maintenance.

---

MAJOR RONALD ROSS, the well-known English authority on the mosquito theory of malaria, has arrived in the United States to visit St. Louis, where he will lecture, and then go to Panama as a guest of the canal commission.

---

THE Berlin Cremation Society has sent to the Pope a petition to which are appended 9,500 signatures, praying that the last rites of the Roman Catholic church shall no longer be denied to persons who wish their remains to be cremated.

---

THE federation of the Catholic Temperance Societies of Belgium has decided to organize an international Congress on Alcoholism to be held in connection with the University Exposition which is to take place at Liege next year.

---

PERSODINE is a mixture of sodium and ammonium persulphates. It is recommended by Bufalini as being superior as an antidote to carbolic acid. The soluble sulphates are the most efficient antidotes to this poison, but these persulphates are more energetic and more quickly effective.

---

A PROCESS for the manufacture of milk flour has recently been devised by a Swede. The product in its dry form is practically imperishable, and when dissolved in water may be used, it is said, for any purpose for which ordinary milk may be used. The cost of reduction to powder is nominal.—*Medical Times.*

---

THE common metallic hairpin may be used as an aesthesiometer, as a probe, as a wire for fractured bones, as a cauterizer, a hairlip pin, a tenaculum, a nasal speculum, a wound retractor, an aneurysm needle, a tracheal retractor, for drainage, a wound approximator, a splinter extractor and a female catheter. The various materials and weights offer differing advantages in different cases. The hairpin is easily sterilized by boiling.

---

JULIA MARLOWE, the actress, announces to the public that she likes her whisky with a little bitter in it. At least, the newspapers are publishing her photograph with the following testimonial: "I am glad to write my endorsement of the great remedy, Peruna, as a nerve tonic. I do so most heartily.—Julia Marlowe." Perhaps it will not be deemed wholly flippant if it is noted that those who take their whisky straight, as a "nerve tonic," do not usually write testimonials thereof.

